# TNF-α enhances Th9 cell differentiation and antitumor immunity via TNFR2-dependent pathways

**DOI:** 10.1186/s40425-018-0494-8

**Published:** 2019-02-04

**Authors:** Yuxue Jiang, Jintong Chen, Enguang Bi, Yinghua Zhao, Tianxue Qin, Yiming Wang, Alison Wang, Sujun Gao, Qing Yi, Siqing Wang

**Affiliations:** 1 Department of Cancer Immunology, The First Hospital of Jilin University, 519 Dongminzhu St, ChangChun, Jilin, China; 2 Department of Cancer Biology, Cleveland Clinic, Lerner Research Institute, Cleveland, OH 44195 USA; 3 Department of Hematology, The First Hospital of Jilin University, Changchun, 130061 China; 4 Department of Orthopedics, China-Japan Union Hospital of Jilin University, Changchun, China; 5 Center for Hematologic Malignancy, Research Institute Houston Methodist Hospital, Houston, TX 77030 USA

**Keywords:** TNF-α, Th9, TNFR2, STAT5

## Abstract

Tumor specific Th9 cells are potential effector cells for adoptive therapy of human cancers. TNF family members OX40L, TL1A and GITRL have been shown to promote the induction of Th9 cells and antitumor immunity. However, the role of TNF-α, the prototype of the TNF superfamily cytokines, in Th9 cell differentiation and their antitumor efficacy is not defined. Here, we showed that TNF-α potently promoted naïve CD4^+^ T cells to differentiate into Th9 cells in vitro. Furthermore, the addition of TNF-α during Th9 cell differentiation increased T cell survival and proliferation. More importantly, the adoptive transfer of TNF-α-treated Th9 cells induced more potent antitumor effects than regular Th9 cells in mouse tumor model. TNF-α signals via two cell surface receptors, TNFR1 and TNFR2. Mechanistic studies revealed that TNF-α drove Th9 cell differentiation through TNFR2 but not TNFR1. In addition, under Th9 polarizing condition, TNF-α activated STAT5 and NF-κB pathways in T cells in a TNFR2-dependent manner. Inhibition of STAT5 and NF-κB pathways by their specific inhibitors impaired TNF-α-induced Th9 cell differentiation. Our results identified TNF-α as a new powerful inducer of Th9 cells and clarified the molecular mechanisms underlying TNF-α-induced Th9 cell differentiation.

## Introduction

Adoptive T-cell therapy (ACT) has shown encouraging results in some cancer types; however, most tumors are refractory to ACT [1]. The efficacy of ACT of cancers relies on the cytolytic activity and the in vivo persistence of the transferred effector T cells [1–4]. Tumor-specific CD8^+^ T cells have powerful cytolytic activities against tumor cells [1]. However, CD8^+^ T cells used in ACT of cancers are often terminally differentiated and have a disappointing lack of persistence in vivo [2]. CD4^+^ T helper (Th) cells are another option of effectors in ACT of cancers. Among Th cell subsets, the cytotoxic Th1 cells are a useful T cell lineage for ACT of cancers [3]. However, Th1 cells display an exhausted phenotype, and have a short-term persistence in vivo [3]. Compared to Th1 cells, the “stem cell-like” memory Th17 cells have reduced cytolytic function in vitro, but persist significantly longer in vivo, resulting in better antitumor efficacy in ACT of cancers [4, 5]. Th9 cells are a unique Th cell subset with a mature T cell phenotype, highly cytolytic activity and prolonged persistence in vivo based on their hyperproliferation, suggesting an excellent effector for ACT of human cancers [6].

Th9 is a new Th cell subset characterized by the secretion of interleukin 9 (IL-9) [7, 8]. Th9 cells can be generated from naïve T cells by the cytokines IL-4 and TGF-β [7, 8]. However, some other cytokines, such as IL-25, TSLP and IL-1β, have been shown to potently stimulate Th9 cell differentiation [9–11]. In addition, multiple transcription factors, such as PU.1, IRF4, and STAT family members STAT1/5/6, have been reported to regulate Th9 cell differentiation [11–15]. In cancer immunology, tumor-specific Th9 cells possess potent antitumor activity and eradicate large tumors in mouse models, better than other Th cell subsets [6, 16, 17]. Various mechanisms may be involved in the antitumor effects of tumor specific Th9 cells. IL-9 enhances effector T cell proliferation [18, 19] and antitumor CTL responses [6, 16]. Th9 cells secrete cytolytic factors, GzmB and GzmA, which mediate direct tumor cytotoxcity [6, 17]. Interestingly, multiple TNF family members, including OX40L, TL1A and GITRL, have been shown to enhance Th9 cell differentiation and their antitumor efficacy [20–23]. However, the role of TNF-α, the prototype member of the TNF superfamily, in Th9 cell development and their antitumor capability is not defined.

TNF-α is a potent proinflammatory cytokine, which is implicated in the immunopathology of various inflammatory diseases [24]. TNF-α stimulates inflammatory cytokine production, cell growth, cell survival and paradoxically, cell death [25–27]. In cancer immunology, TNF-α activates antigen-presenting cells and promotes the activation and proliferation of effector T cells [28, 29]. TNF-α impairs the function of regulatory T (Treg) cells [30], which contributes to antitumor immunity. TNF-α has two cell surface receptors TNFR1 and TNFR2 [24]. TNFR1 is widely expressed, whereas the expression of TNFR2 is limited to immune and endothelial cells [24, 31]. The cytoplasmic regions of TNFR1 contain a conserved ‘death’ domain which is essential for triggering cell apoptosis and subsequent activation of NF-κB [27, 31]. In contrast, TNFR2 lacks the cytoplasmic ‘death’ domain and has mainly been linked to cell survival and proinflammatory reactions [31, 32].

In this study we found that TNF-α profoundly stimulates Th9 cell differentiation. And the adoptive transfer of TNF-α-treated Th9 cells induces more potent inhibition on melanoma tumor growth than regular Th9 cells in mouse models. In addition, we clarified the TNFR2-dependent signaling pathways of TNF-α-induced Th9 cell differentiation.

## Materials and methods

### Mice and cell lines

C57BL/6 (H-2^b^), TNFR1^−/−^ (B6.129-Tnfrsf1a^tm1Mak^/J) and TNFR2^−/−^ (B6.129S2-Tn frsf1b^tm1Mwm^/J) mice were purchased from the Jackson Laboratory. Mice were housed in specific pathogen-free conditions at the First Hospital Animal Center of Jilin University. Mice at 6–8 weeks of age were used in experiments. All animal experiments were approved by the Animal Ethical Committee of First Hospital of Jilin University.

B16 and B16-OVA melanoma cells were purchased from ATCC (Rockville, MD). Cells were cultured in RPMI 1640 medium supplemented with 10% heat-inactivated fetal bovine serum (FBS, Hyclone), 100 U/mL penicillin and 100 mg/mL streptomycin (both from Invitrogen).

### Reagents

Recombinant mouse IL-4, TNF-α and human TGF-β were purchased from R&D Systems. CFSE (carboxylfluorescein diacetate, succinimidyl ester) was purchased from Invitrogen. Functional anti-mouse CD3e and CD28 antibodies (mAbs) were purchased from eBioscience. Anti-TNFR1 and anti-TNFR2 blocking mAbs and control IgG were purchased from Biolegend. STAT5 inhibitor and Bortezomib (a NF-κB inhibitor) were purchased from Santa Cruz and Selleckchem respectively.

### In vitro Th9 cell differentiation

Naive CD4^+^ T cells (CD4^+^CD25^−^CD62L^hi^) were purified from spleen cells by fluorescence activated cell sorter (FACS). Naïve CD4^+^ T cells (1 × 10^5^ per well) were cultured in the presence of plate-bound anti-CD3 (2 μg/mL) plus soluble anti-CD28 (2 μg/mL) and Th9-polarizing cytokines TGF-β (3 ng/mL) and IL-4 (10 ng/mL). Cells from cultures without addition of TGF-β and IL-4 were used as Th0 cells. In some cell cultures, TNF-α (50 ng/mL) was added. After 3 days of culture, the cells and culture supernatants were harvested and analyzed by flow cytometry, ELISA and/or qPCR.

To examine the role of TNFR1 and TNFR2 in TNF-α-induced Th9 cell differentiation, naïve CD4^+^ T cells were cultured under the Th9-polarizing conditions with or without addition of TNF-α (50 ng/mL). Cell cultures were added with anti-TNFR1 (50 μg/mL), anti-TNFR2 (50 μg/mL) mAbs or control IgG (50 μg/mL). After 3 days of culture, the cells and culture supernatants were harvested and analyzed.

To explore the signaling pathways involved in TNF-α-induced Th9 cell differentiation, naïve CD4^+^ T cells were cultured under the Th9-polarizing conditions in the presence or absence of TNF-α. In some cell cultures, STAT5 Inhibitor (10 μg/mL) or Bortezomib (1 nM) was added. After 3 days of culture, the cells and culture supernatants were harvested and analyzed.

### Flow cytometry

Flow cytometry analysis was performed as described previously [23]. PE-Cy7-, FITC-, or Alex Fluor 700-conjugated mAbs against CD4 (cat #: 552775), CD25 (cat #: 553072), CD62L (cat #: 560517) and CD44 (cat #:561859) were purchased from BD Biosciences. PE- or APC-conjugated mAbs against IL-9 (cat #: 514103), TNFR1 (cat #: 113005) and TNFR2 (cat #: 113405) were purchased from Biolegend. Intracellular staining was performed by using a Cytofix/Cytoperm kit (BD Biosciences) according to the manufacturer’s instruction. Cells were acquired and analyzed by a BD LSRFortessa™ cytometer.

### Quantitative polymerase chain reaction (qPCR)

Cellular RNA was extracted with the EasyPure RNA Kit (TransGen Biotech) and cDNA was amplified with an All-in-One First-Strand cDNA Synthesis SuperMix (TransGen Biotech). The expression of *Il9*, *Ifng*, *Il4*, *Il5*, *Il13*, *Il17*, *Spi1*, *Irf4*, *Tbx21, Gata3*, *Rorc* and *Foxp3* by Th cells were analyzed with SYBR Green real-time PCR (Applied Biosystems). Gene expression was normalized to *Gapdh*. Primer sets were shown in the previous publication [23].

### Western-blot analyses

Western-blot assay was performed as previously described [23]. Anti-mouse phosphorylated (p)-STAT1, p-STAT3, p-STAT5, p-STAT6, p-IKKα/β, IκB-α and β-actin antibodies were purchased from Cell Signaling Technology (CST). p-STAT2 was purchased from Abcam. And p-STAT4 was purchased from Invitrogen.

### Enzyme-linked immunosorbent assay (ELISA)

Concentrations of IL-9 in culture supernatant were detected by ELISA as previously described [23]. IL-9 Capture/detection antibodies were purchased from BD Biosciences. Recombinant mouse IL-9 used as the standards in ELISA were purchased from R&D Systems. Avidin-HRP was purchased from Biolegend.

### RNA sequencing (RNA-Seq)

Mouse naïve CD4^+^ T cells were cultured under Th9 polarizing conditions with or without addition of TNF-α for 6 h and cells were collected for RNA extraction. Total RNA was extracted with the Trizol (ThermoFisher) and RNA-Seq was done by the genomics core of Lerner Research Institute in Cleveland Clinic with Illumina HiSeq2500.

### Co-immunoprecipitation (co-IP) and mass spectrometry (MS)

Naïve CD4^+^ T cells were cultured under Th9 polarizing conditions with or without addition of TNF-α for 3 h. Cells were collected and cell lysates were prepared in non-denaturing lysis buffer. Cell lysates were incubated with anti-TNFR2 antibody for 2 h at 4 °C, and subjected to immunoprecipitation (IP) using protein A-sepharose beads. The beads were washed with IP buffer. The proteins were eluted with SDS sample buffer and heated at 98 °C for 5 min. IP samples were then separated using SDS-PAGE and visualized by Coomassie Blue staining. The immunoreactive bands were excised from stained gels and digested overnight with trypsin (10 ng/μL) at room temperature. Peptides in the digested sample were analyzed using liquid chromatography mass spectrometry (LC-MS) provided by Mass Spectrometry Laboratory for Protein Sequencing (Lerner Research Institute, Cleveland Clinic, Cleveland, Ohio, USA).

### Luciferase reporter assays

The luciferase reporter vector pGL4.10, a control vector pGL4.74 and expression vectors for NF-κB molecules p50, p65, c-Rel, p52 and RelB were purchased from Addgene. A 2500-bp mouse *Il9* promoter was inserted into pGL4.10 (mIl9-pGL4.10). HEK293T cells were transiently transfected with mIl9-pGL4.10 (0.25 μg per well), or pGL4.74 (0.05 μg per well) and expression vectors (0.5 μg per well) for NF-κB molecules by Lipofectamine 2000 (Invitrogen). Promoter activity was measured with Dual-Luciferase Reporter Assay System (Promega) according to the manufacturer’s instructions. Values are normalized to internal control and expressed as the Mean ± SD of relative luciferase units.

### Adoptive tumor immunotherapy

2 × 10^5^ B16-OVA cells were injected subcutaneously into C57BL/6 mice. To generate Th9 cells, naïve CD4^+^ T cells from OT-II mice were cultured under Th9 polarizing conditions in the presence or absence of TNF-α for 2 days. On Day 2 after tumor injection, the mice were randomly divided into groups and transfused with Th9 or TNF-α-treated Th9 cells (1 × 10^6^) via tail vein injection. Mice treated with PBS served as controls. Tumor development was monitored over time. The mice were killed when the tumor diameter reached between the range of 1.5 and 2 cm. Tumor volume was calculated by the formula: 3.14 × (mean diameter)^3^/6.

### Statistical analysis

The Student t test (2 groups) and one-way ANOVA (> = 3 groups) were used to compare various experimental groups. A *P* value of less than 0.05 was considered significant.

## Results

### TNF-α promotes Th9 cell differentiation in vitro

**Fig. 1:**
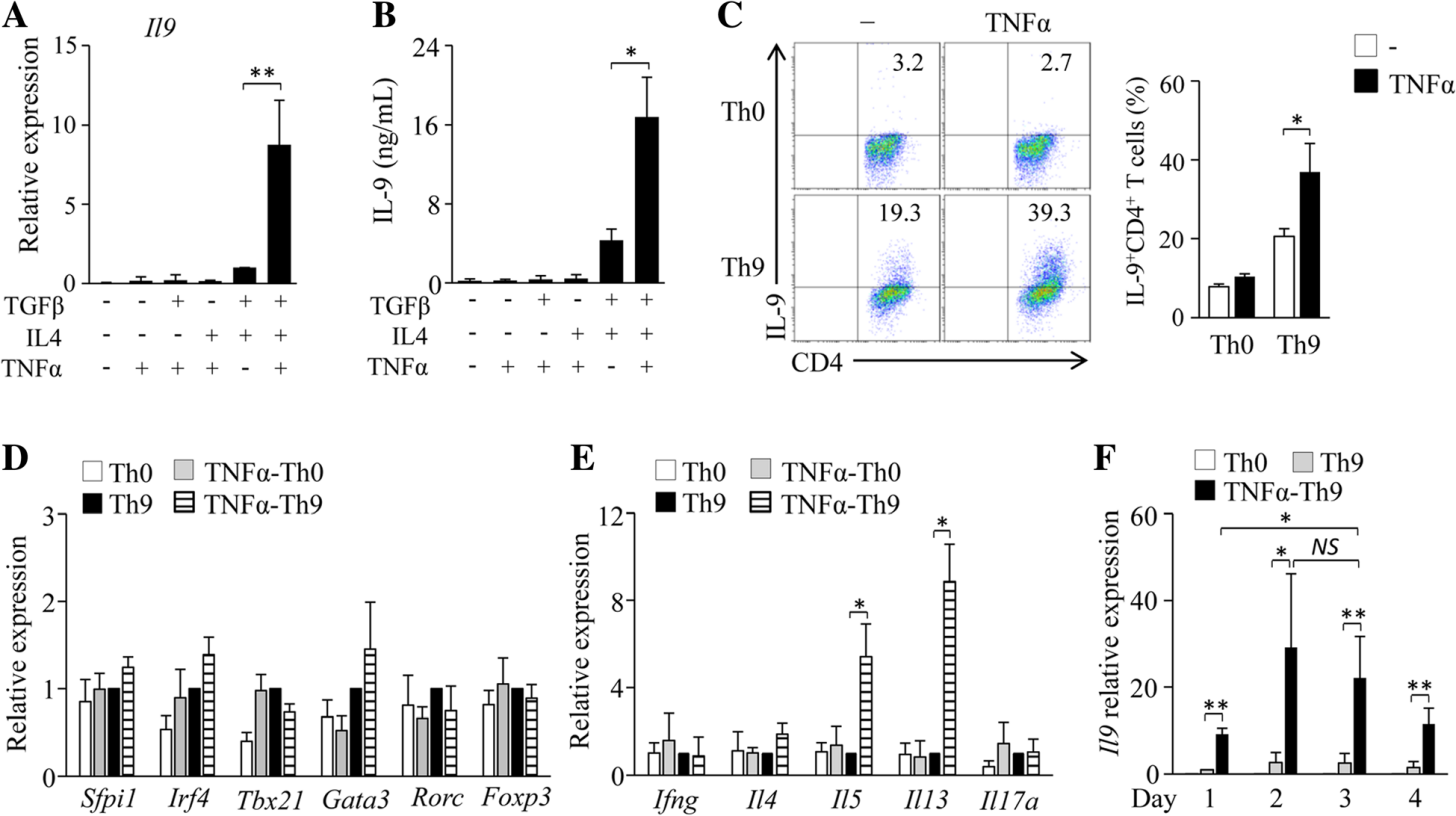
TNF-α drives Th9 cell differentiation in vitro. (**a**, **b**) Mouse naïve CD4^+^ T cells were cultured in the presence of anti-CD3/28 with the addition of TGF-β, IL-4, TNF-α or their combinations for 3 days. Cultures without the addition of any cytokines were used as controls. (**a**) qPCR analysis of *Il9* gene expression in CD4^+^ T cells. Expression was normalized to *Gapdh* and set at 1 in cells treated with TGF-β plus IL-4 (Th9 cells). (**b**) ELISA assessment of IL-9 secretion in the cultures. (**c**-**e**) Naïve CD4^+^ T cells were cultured under Th9 polarizing conditions with or without addition of TNF-α for 3 days. Cell cultures without (Th0) addition of Th9-polarizing cytokines TGF-β and IL-4 were used as controls. (**c**) Flow cytometry analysis of IL-9-expressing CD4^+^ (IL-9^+^CD4^+^) T cells. Numbers in the dot plots represent the percentages of IL-9^+^CD4^+^ T cells. Right, summarized results of three independent experiments obtained as at left. (**d**, **e**) qPCR analysis of the indicated transcription factors (**d**) and cytokines (**e**). (**f**) Naïve CD4^+^ T cells were cultured under Th9 polarizing conditions in the presence or absence of TNF-α. Th0 cells were used as controls. Cells were collected at the indicated time points and qPCR analyzed the expression of *Il9* in CD4^+^ T cells. Expression was normalized to *Gapdh* and set at 1 in Th9 cells collected on Day 1. Expression was normalized to *Gapdh* and set at 1 in Th9 cells. Data are representative of three (**c**) independent experiments or presented as mean ± SD of three (**a**-**f**) independent experiments. *NS*, non-significant; *P < 0.05; **P < 0.01

To examine the role of TNF-α in Th9 cell differentiation, naïve CD4^+^ T cells were cultured in the presence of anti-CD3/28 antibodies plus TGF-β, IL-4 and/or TNF-α for 3 days. The addition of TNF-α combined with Th9 polarizing cytokines TGF-β and IL-4 increased Th cell expression of IL-9 mRNA and protein (Fig. 1a, b), and the frequency of Th9 cells (Fig. 1c). However, TNF-α alone or TNF-α plus TGF-β or IL-4 could not induce Th9 cell differentiation (Fig. 1a-c). Interestingly, TNF-α did not increase the expression of *Spi1* or *Irf4* in Th9 cells (Fig. 1d), suggesting that TNF-α may drive Th9 cell differentiation through other Th9-related transcription factors. We also examined the expression of the other Th cell-related cytokines and transcription factors and found that TNF-α-treated Th9 cells did not express most of Th1-, Th2-, Th17- and Treg-related cytokines and transcription factors, such as *Ifng*, *Il4*, *Il17*, *Tbx21*, *Gata3*, *Rorc* and *Foxp3* (Fig. 1d, e), although *Il5* and *Il13* were increased (Fig. 1e) in TNF-α-treated Th9 cells compared to regular Th9 cells. We also examined the effects of TNF-α on the expression of *Il9* in Th9 cells at different time points. We found that the expression of *Il9* in TNF-α-treated Th9 cells increased on Day 1, reached the highest level on Day 2 or Day 3, and then slightly decreased from the highest level on Day 4 (Fig. 1f). Together, these results demonstrated that TNF-α promotes Th9 cell differentiation in vitro.

### TNF-α increases the survival and proliferation of Th9 cells in vitro

**Fig. 2:**
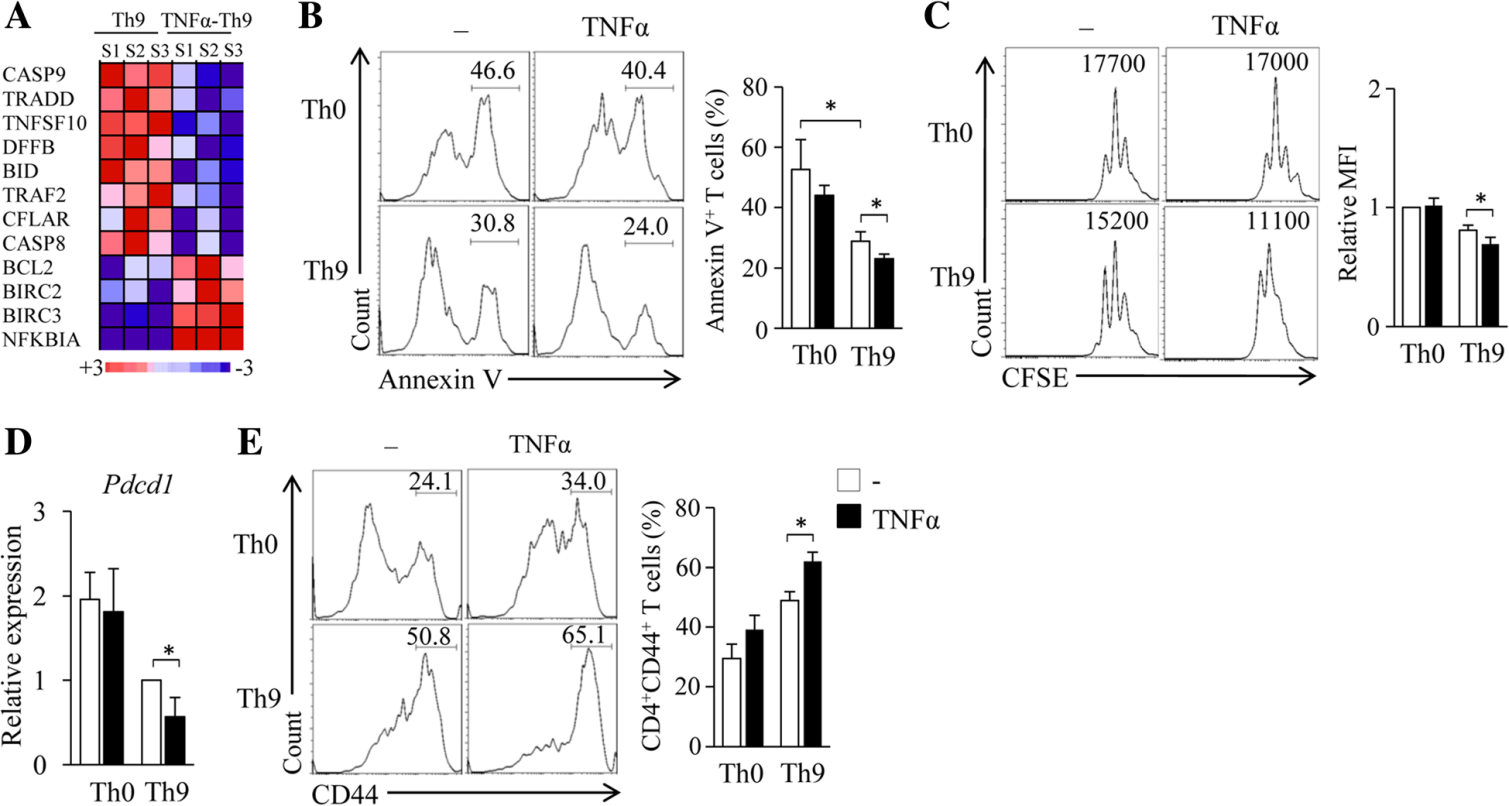
TNF-α increases the survival and proliferation of Th cells in vitro. (**a**) Mouse naïve CD4^+^ T cells were cultured under Th9 polarizing conditions with or without the addition of TNF-α for 6 h. The experiments were repeated three times. Cell samples (S1–3) were analyzed by RNA-seq. Pink-blue heatmap shows the log_2_-fold change of the differentially regulated expression of genes related to cell death pathways. Pink, higher expression; blue, lower expression. (**b**) Mouse naïve CD4^+^ T cells were cultured under Th0 or Th9 polarizing conditions in the presence or absence of TNF-α for 3 days. Flow cytometry analyzed Annexin V^+^ T cells. Numbers in the histograms represent the percentages of Annexin V^+^ T cells. Right, summarized results of three independent experiments obtained as the left. (**c**) Naïve CD4^+^ T cells were labeled with CFSE and cultured under Th0 or Th9 polarizing conditions in the presence or absence of TNF-α for 3 days. Flow cytometry analyzed CFSE-stained T cells. Numbers in the histograms represent the fluorescence intensity (FI) of CFSE-stained T cells. Right, summarized results of three independent experiments obtained as the left. MFI, mean fluorescence intensity. (**d**, **e**) Naïve CD4^+^ T cells were cultured as shown in (**b**). (**d**) qPCR assessed the expression of *Pdcd1* in cultured T cells. (**e**) Flow cytometry analyzed the expression of CD44 by T cells. Numbers in the histograms represent the percentages of CD44^+^ T cells. Right, summarized results of three independent experiments obtained as the left. Data are representative of three (**b**, **c**,** e**) independent experiments or presented as mean ± SD of three (**b**-**e**) independent experiments. *P < 0.05

The survival and proliferation of tumor-specific T cells are crucial for their persistence and therapeutic potential in vivo. We next examined the effects of TNF-α treatment on Th9 cell survival and proliferation. Naïve CD4^+^ T cells were cultured under Th9-polarizing conditions in the presence or absence of TNF-α for 6 h. RNA-seq analysis of the cultured T cells revealed that the addition of TNF-α decreased T cell expression of *Casp9*, *Tradd*, *Tnfsf10*, *Dffb*, *Bid*, *Traf2*, *Cflar* and *Casp8* (Fig. 2a), the genes related to cell apoptosis, and increased the expression of *Bcl2*, *Birc2*, *Birc3* and *Nfkbia* (Fig. 2a), the genes related to cell survival, suggesting that the addition of TNF-α during Th9 cell differentiation inhibits T cell apoptosis and promotes T cell survival.

To further exploit the role of TNF-α in Th9 cell survival and proliferation, Th9 cells with or without TNF-α-treatment were generated and cell death was assessed by Annexin V staining. While Th9 cells showed less cell apoptosis than Th0 cells (Fig. 2b), TNF-α treatment further reduced the apoptosis of Th9 cells (Fig. 2b). In addition, TNF-α treatment increased Th9 cell proliferation (Fig. 2c). These results demonstrated that TNF-α enhances T cell survival and proliferation during Th9 cell differentiation.

PD-1 is an immune-checkpoint and PD-1 up-regulation promotes CD4^+^ T cell apoptosis [33]. We next examined the role of TNF-α on the expression of PD-1 by Th9 cells. While Th9 cells expressed lower mRNA levels of *Pdcd1* than Th0 cells (Fig. 2d), TNF-α treatment further decreased the expression of *Pdcd1* in Th9 cells (Fig. 2d). Furthermore, TNF-α treatment increased Th9 cell expression of CD44 (Fig. 2e), a marker for effector and memory T cells which contribute to T cell survival and proliferation [34]. Together, these results demonstrated that TNF-α enhances Th9 cell survival and proliferation in vitro*.*

### TNF-α treatment improves the antitumor efficacy of Th9 cells in vivo

**Fig. 3:**
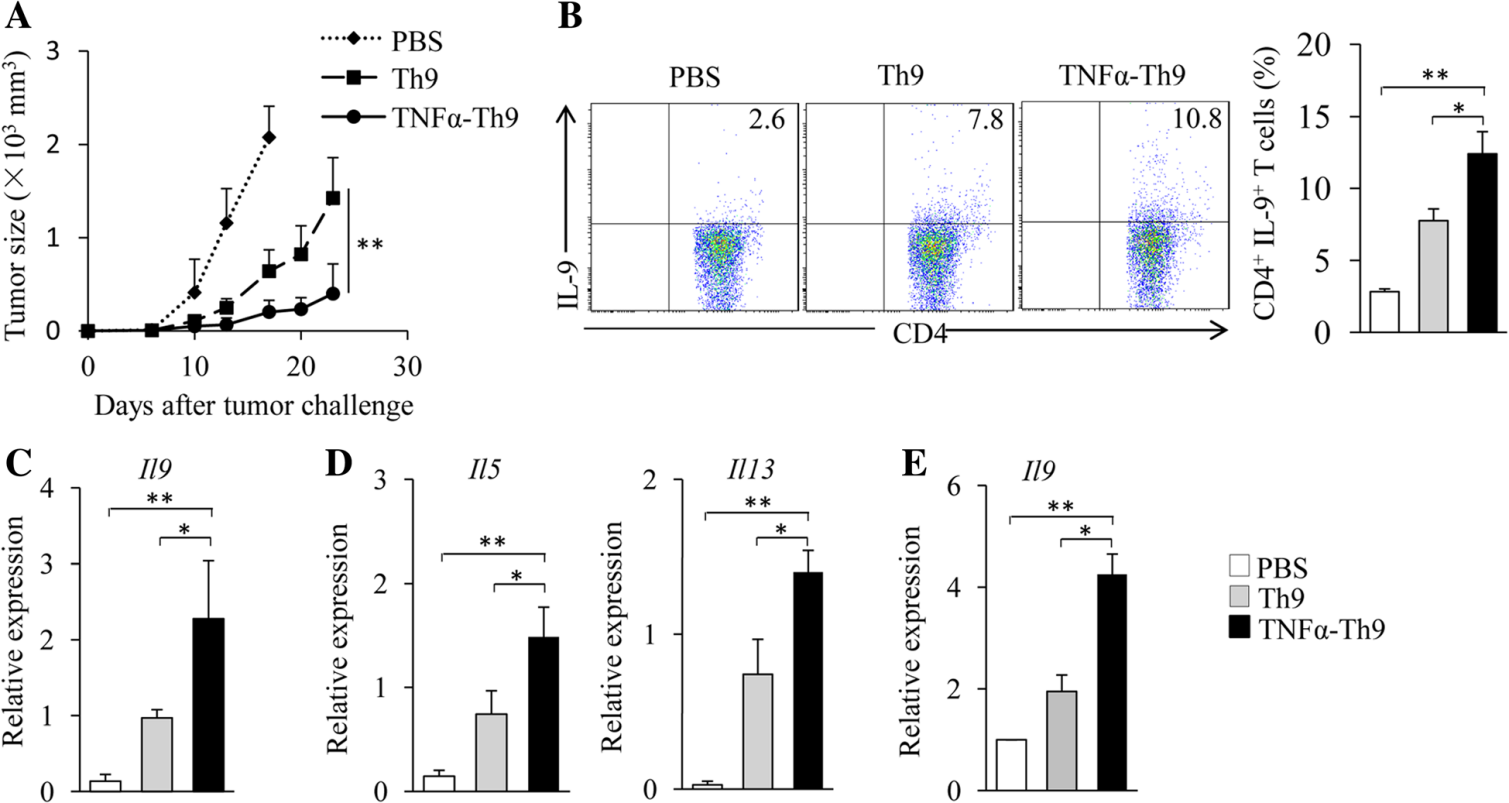
TNF-α-treated Th9 cells exhibit increased antitumor efficacy in vivo. (**a**) Naïve CD4^+^ T cells from OT-II mice were cultured under Th9 polarizing conditions with or without the addition of TNF-α for 2 days. C57BL/6 mice (five mice/group) were injected s.c. with 2 × 10^5^ B16-OVA cells. On Day 2 after tumor challenge, Th9 or TNF-α-treated Th9 cells (1 × 10^6^) were injected i.v. into the B16-OVA tumor-bearing mice. Mice treated by PBS served as controls. Shown are the tumor growth curves. The experiments were performed twice with a total of 10 mice per group (*n* = 10). (**b**-**d**) C57BL/6 mice were injected s.c. with 5 × 10^5^ B16-OVA cells. OT-II Th9 cells and TNF-α-treated Th9 cells were generated in vitro as in (A). On Day 5 after tumor challenge, mice (*n* = 3/group) were given i.v. with Th9 or TNF-α-treated Th9 cells (3 × 10^6^) or PBS control. On Day 3 after T cell transfusion, cells were isolated from tumor-draining lymph nodes (TDLNs). (**b**) Flow cytometry analysis of IL-9-producing CD4^+^ T cells. Numbers in the dot plots represent the percentages of double-positive T cells. Right, summarized results of three independent experiments obtained as at left. (**c**, **d**) CD4^+^ T cells were isolated from TDLNs by MACS. qPCR examined the expression of *Il9* (**c**), *Il5* and *Il13* (**d**) in CD4^+^ T cells. (**e**) C57BL/6 mice were injected i.v. with 5 × 10^5^ B16-OVA cells. OT-II Th9 and TNF-α-treated Th9 cells were generated in vitro as in (**a**). On Day 5 after tumor challenge, mice (*n* = 3/group) were given i.v. with Th9 or TNF-α-treated Th9 cells (3 × 10^6^) or PBS control. On Day 3 after T cell transfusion, CD4^+^ T cells were separated from the lung tumor tissues by MACS. qPCR assessed the expression of *Il9* in CD4^+^ T cells. Data are representative of three (**b**) independent experiments or presented as mean ± SD of three (**b**-**e**) independent experiments. *P < 0.05; **P < 0.01

To assess the antitumor efficacy of TNF-α-treated Th9 cells, naïve CD4^+^ T cells from OT-II mice were differentiated into Th9 cells in the presence or absence of TNF-α. Cells were used to treat B16-OVA-bearing C57BL/6 mice. While Th9 cells mediated a higher inhibition on melanoma tumor growth than PBS control (Fig. 3a), the addition of TNF-α during Th9 cell differentiation further improved their antitumor efficacy (Fig. 3a), demonstrating that TNF-α improves the antitumor efficacy of Th9 cells.

The infiltration of effector T cells into tumor sites is associated with their antitumor effects. We next examined the tumor-infiltrating capability of TNF-α-treated Th9 cells. OT-II Th9 cells and TNF-α-treated Th9 cells were transfused to B16-OVA-bearing C57BL/6 mice, and cells from tumor-draining lymph nodes (TDLNs) were collected and analyzed. While mice transfused with Th9 cells had higher frequencies of IL-9^+^CD4^+^ T cells in TDLNs than PBS control mice (Fig. 3b), the percentages of IL-9^+^CD4^+^ T cells in TDLNs further increased in mice receiving TNF-α-treated Th9 cells compared to regular Th9 cells (Fig. 3b). In addition, TDLN CD4^+^ T cells from mice receiving TNF-α-treated Th9 cells expressed higher levels of *Il9* (Fig. 3c), *Il5* and *Il13* (Fig. 3d) than mice transfused with Th9 cells or PBS control. We also examined CD4^+^ T cells isolated from pulmonary tumor tissues. As shown in Fig. 3e, cells from mice receiving TNF-α-treated Th9 cells expressed higher levels of *Il9* than mice transfused with Th9 cells or PBS controls. These results indicated that TNF-α treatment increases the tumor-infiltrating capability of Th9 cells.

### TNF-α enhances Th9 cell differentiation through TNFR2

**Fig. 4:**
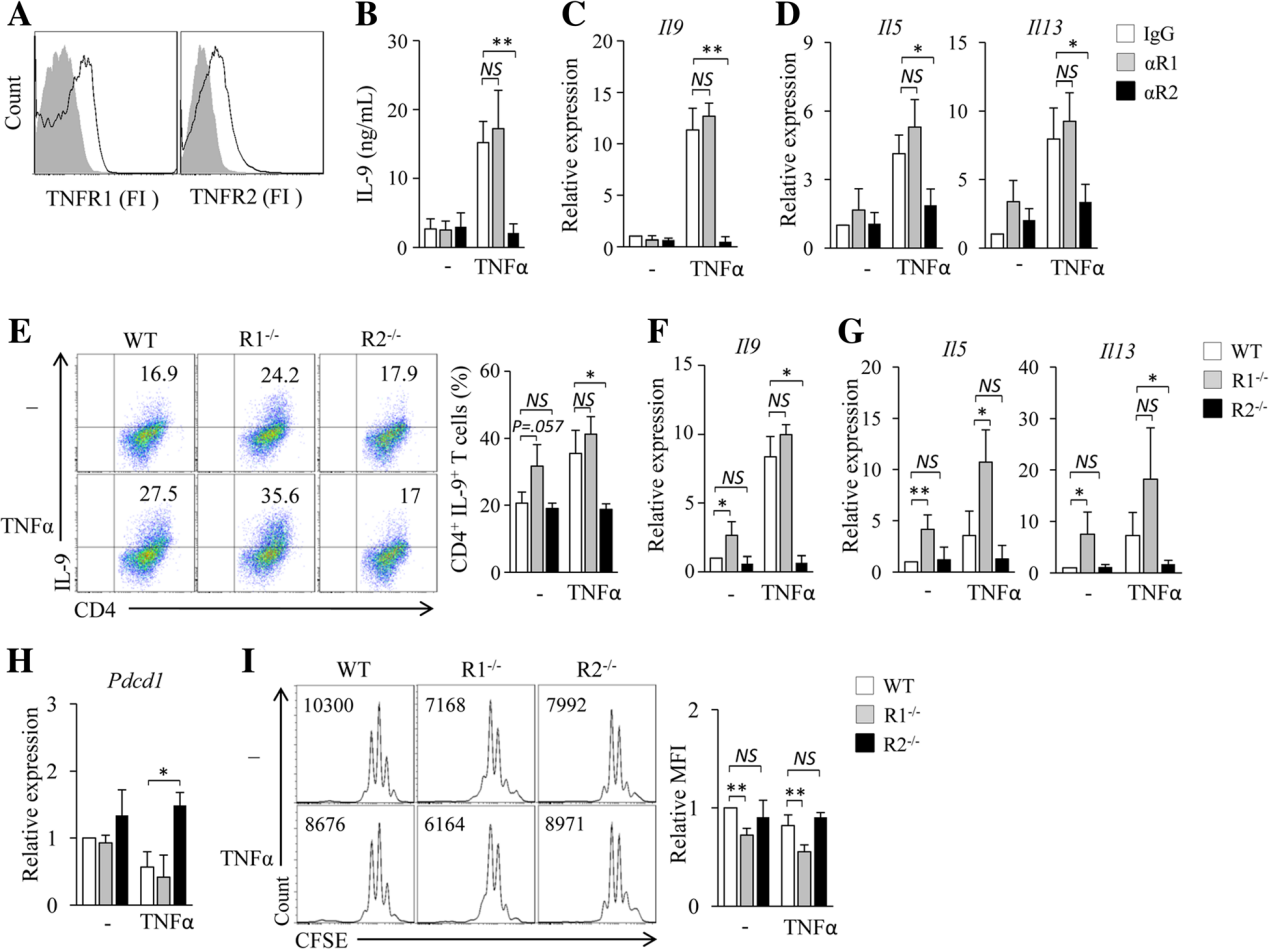
TNF-α enhances Th9 cell differentiation through TNFR2 but not TNFR1. (**a**) Flow cytometry examined analysis of TNFR1 and TNFR2 in mouse CD4^+^ T cells. (**b**-**d**) CD4^+^ naïve T cells were cultured under Th9 polarizing conditions in the presence of TNFR1 (αR1) or TNFR2 (αR2) blocking antibodies or an isotype control IgG (IgG) with or without (−) addition of TNF-α for 3 days. (**b**) ELISA assessed IL-9 secretion in the culture. (**c**, **d**) qPCR assessed the expression of *Il9* (**c**) and *Il5* and *Il13* (**d**) in CD4^+^ T cells. (E-H) Naïve CD4^+^ T cells were isolated from wild type (WT), TNFR1^−/−^ (R1^−/−^) or TNFR2^−/−^ (R2^−/−^) mice and cultured under Th9 polarizing conditions with or without addition of TNF-α for 3 days. (**e**) Flow cytometry analysis of IL-9^+^CD4^+^ T cells. Numbers in the dot plots represent the percentages of IL-9^+^CD4^+^ T cells. Right, summarized results of three independent experiments obtained as at left. (**f**, **g**) qPCR analysis of *Il9* (**f**), *Il5* and *Il13* (**g**) in CD4^+^ T cells. (**h**) qPCR analysis of *Pdcd1* in T cells. (**i**) Naïve CD4^+^ T cells from WT, R1^−/−^ and R2^−/−^ mice were labeled with CFSE and cultured under Th9 polarizing conditions with or without the addition of TNF-α for 3 days. Flow cytometry analyzed CFSE-stained T cells. Numbers in the histograms represent the fluorescence intensity of CFSE-stained T cells. Right, summarized results of three independent experiments obtained as the left. Data are representative of three (**a**, **e**, **i**) independent experiments or presented as mean ± SD of three (**b**-**i**) independent experiments. *NS*, non-significant; *P < 0.05; **P < 0.01

There are two cell surface receptors for TNF-α, TNFR1 and TNFR2 [24]. CD4^+^ T cells expressed both TNFR1 and TNFR2 (Fig. 4a). We next explored the contribution of TNFR1 and TNFR2 to TNF-α-induced Th9 cell differentiation. TNFR1 (αR1) or TNFR2 (αR2) blocking antibodies were used during the in vitro differentiation of Th9 cells with or without the addition of TNF-α. The addition of αR1 did not affect the expression of IL-9 mRNA and protein in TNF-α-treated Th9 cells as compared to control IgG (Fig. 4b, c), whereas the addition of αR2 abolished TNF-α-induced up-regulation of IL-9 expression in Th9 cells (Fig. 4b, c). In addition, the addition of αR2 but not αR1 abolished the up-regulation of *Il5* and *Il13* expression induced by TNF-α in Th9 cells (Fig. 4d). These results indicated that TNFR2 but not TNFR1 mediates the stimulatory activity of TNF-α in Th9 cell differentiation.

To further confirm the functional role of TNFR1 and TNFR2 in TNF-α-induced Th9 cell differentiation, naïve CD4^+^ T cells were isolated from WT, R1^−/−^ and R2^−/−^ mice and differentiated into Th9 cells in the presence or absence of TNF-α. As compared to WT cells, TNFR1-deficiency displayed minor effects on or even slightly increased TNF-α-induced Th9 cell production and *Il9* expression by R1^−/−^ Th9 cells (Fig. 4e, f). However, the capability of TNF-α in promoting R2^−/−^ Th9 cell differentiation was completely abolished as demonstrated by significantly lower cell frequencies of R2^−/−^ Th9 cells and lower *Il9* expression by R2^−/−^ Th9 cells as compared to TNF-α-treated WT Th9 cells (Fig. 4e, f). Notably, as compared to WT cells, R1^−/−^ cells developed into more IL-9-expressing Th9 cells and R1^−/−^ Th9 cells expressed higher levels of *Il9* mRNA (Fig. 4e, f) in the cultures without the addition of TNF-α, indicating that the endogenously produced TNF-α reinforced R1^−/−^ Th9 cell differentiation and suggesting that the TNF-α/TNFR1 signaling may counteract the signaling of TNF-α/TNFR2 in the induction of Th9 cells. Similarly, the deficiency of TNFR2 but not TNFR1 abolished the up-regulation of *Il5* and *Il13* expression in both Th9 cells and TNF-α-treated Th9 cells (Fig. 4g). Interestingly, while TNF-α-treated R1^−/−^ Th9 cells had comparable expression of *Pdcd1* as compared to TNF-α-treated WT Th9 cells (Fig. 4h), TNFR2 deficiency abolished TNF-α-induced inhibition of *Pdcd1* expression in Th9 cells (Fig. 4h). Furthermore, the deficiency of TNFR2 but not TNFR1 abrogated TNF-α-induced stimulation of Th9 cell proliferation (Fig. 4i). Collectively, these results demonstrated that TNF-α stimulates Th9 cell differentiation through TNFR2 but not TNFR1.

### TNF-α enhances Th9 cell differentiation through TNFR2-STAT5 signaling

**Fig. 5:**
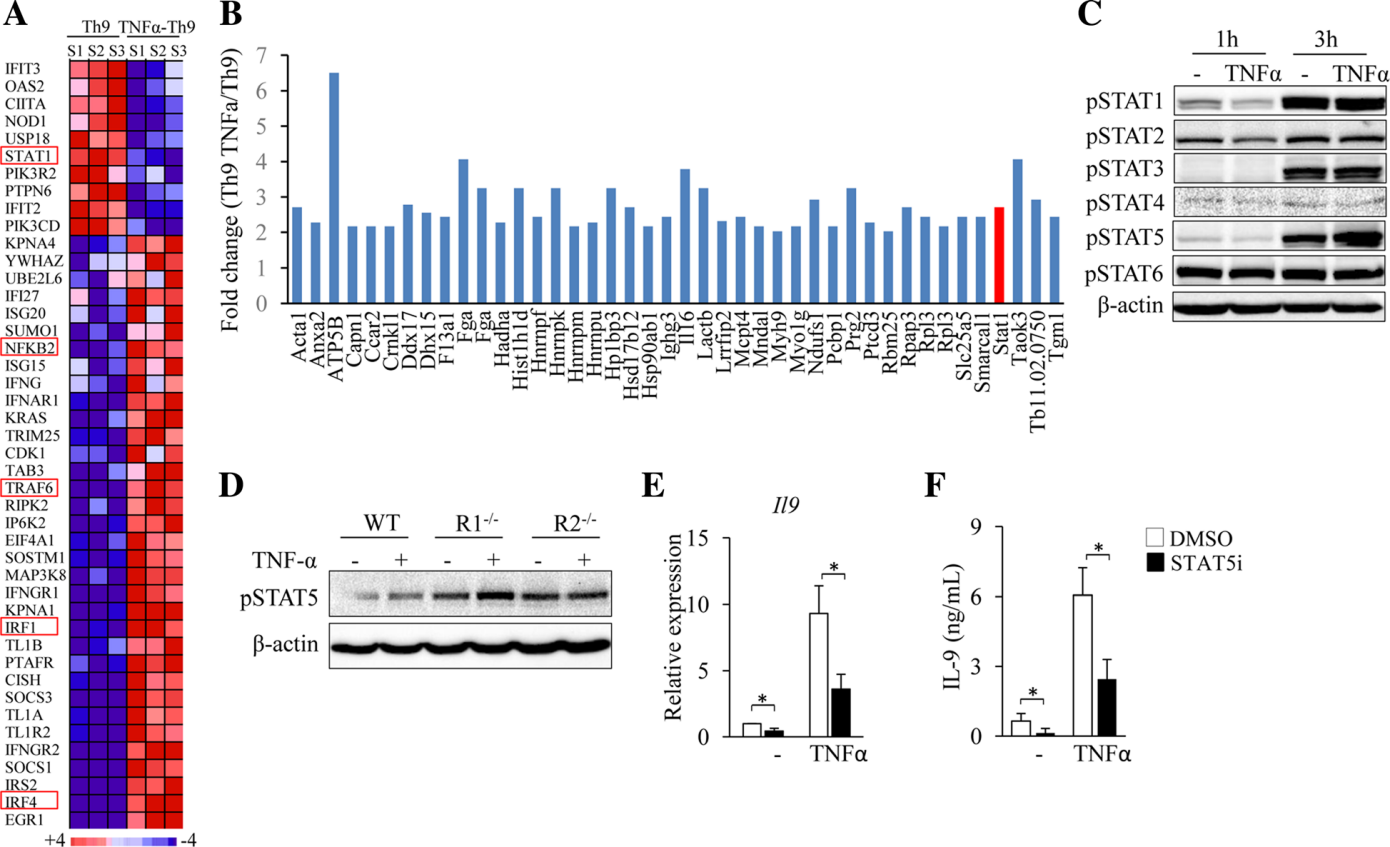
TNF-α enhances Th9 cell differentiation through STAT5. (**a**) The RNA-seq data mentioned in Fig. 2a were used. Pink-blue heatmap shows the log_2_-fold change of the differentially expressed genes of signaling pathways that are potentially involved in TNF-α-induced Th9 cell differentiation. (**b**) Naïve CD4^+^ T cells were cultured under Th9 polarizing conditions with or without addition of TNF-α for 3 h. Immunoprecipitation (IP) was performed by using anti-TNFR2 and proteins in the immune precipitate were analyzed by mass spectrometry (MS). Shown are the fold changes (TNF-α-Th9/Th9) of proteins with 2-fold cut-off. (**c**) Naïve CD4^+^ T cells were cultured under Th9 polarizing conditions with or without addition of TNF-α for 1 or 3 h. Western-blots examined the protein levels of the phosphorylated STAT family members (pSTAT1–6). β-actin was used as a loading control. (**d**) Naïve CD4^+^ T cells from WT, R1^−/−^ or R2^−/−^ mice were cultured under Th9 polarizing conditions with or without the addition of TNF-α for 3 h. Western-blots analyzed pSTAT5 and β-actin in T cells. (**e**, **f**) Naïve CD4^+^ T cells were cultured under Th9 polarizing conditions in the presence or absence of TNF-α with or without (DMSO) addition of STAT5 inhibitor (STAT5i) for 3 days. IL-9 expression was examined by qPCR (**e**) and ELISA (**f**). Data are representative of three (**c**, **d**) independent experiments or presented as mean ± SD of three (**e**, **f**) independent experiments. *P < 0.05

We next exploited the downstream signaling pathways of TNFR2 that are responsible for TNF-α-induced Th9 cell differentiation. RNA-seq assay showed that the addition of TNF-α during Th9 cell differentiation decreased the expression of *Stat1* and increased the expression of *Nfkb2*, *Traf6*, *Irf1* and *Irf4* (Fig. 5a), genes related to cytokine-induced signaling pathways. To exploit the proteins that may interact with TNFR2, naïve CD4^+^ T cells were cultured under Th9 polarizing conditions in the presence or absence of TNF-α and immunoprecipitation (IP) of the cell lysates was performed by using an anti-TNFR2 antibody, followed by mass spectrometry (MS) analysis of the immune precipitates. Interestingly, higher levels of STAT1 were detected in TNF-α-treated cells as compared to untreated control cells (Fig. 5b), suggesting that STAT signaling pathways may be involved TNF-α-induced Th9 cell differentiation.

We next explored the effects of TNF-α on the activation of STAT family members in T cells cultured under Th9 polarizing condition. Western-blots detected increased levels of phosphorylated (p) STAT1 (pSTAT1), pSTAT3 and pSTAT5 in T cells at Hour 3 compared to Hour 1 (Fig. 5c); whereas only pSTAT5 increased further in TNF-α-treated T cells compared to untreated controls at Hour 3 (Fig. 5c). These results indicated that TNF-α enhances STAT5 activation during Th9 cell differentiation.

To determine the role of TNFR1 and TNFR2 in TNF-α-induced activation of STAT5, naïve CD4^+^ T cells were isolated from WT, R1^−/−^ and R2^−/−^ mice and cultured under Th9 polarizing conditions in the presence or absence of TNF-α. The addition of TNF-α remarkably increased pSTAT5 in R1^−/−^ T cells as compared to the untreated control (Fig. 5d); however, TNF-α treatment exhibited minor effects on the protein levels of pSTAT5 in R2^−/−^ T cells as compared to the untreated control (Fig. 5d). These results demonstrated that TNF-α activates STAT5 via TNFR2 but not TNFR1.

To investigate the role of STAT5 signaling in TNF-α-induced Th9 cell differentiation, a STAT5 specific inhibitor (STAT5i) was used during Th9 cell differentiation. The inhibition of STAT5 significantly decreased IL-9 mRNA and protein expression in both TNF-α-treated Th9 cells and regular Th9 cells (Fig. 5e, f), indicating that TNF-α-induced Th9 cell differentiation relies on STAT5 signaling pathways. Collectively, these results demonstrated that TNF-α induces Th9 cell differentiation through TNFR2-mediated activation of STAT5.

### TNF-α enhances Th9 cell differentiation through NF-κB pathway

**Fig. 6:**
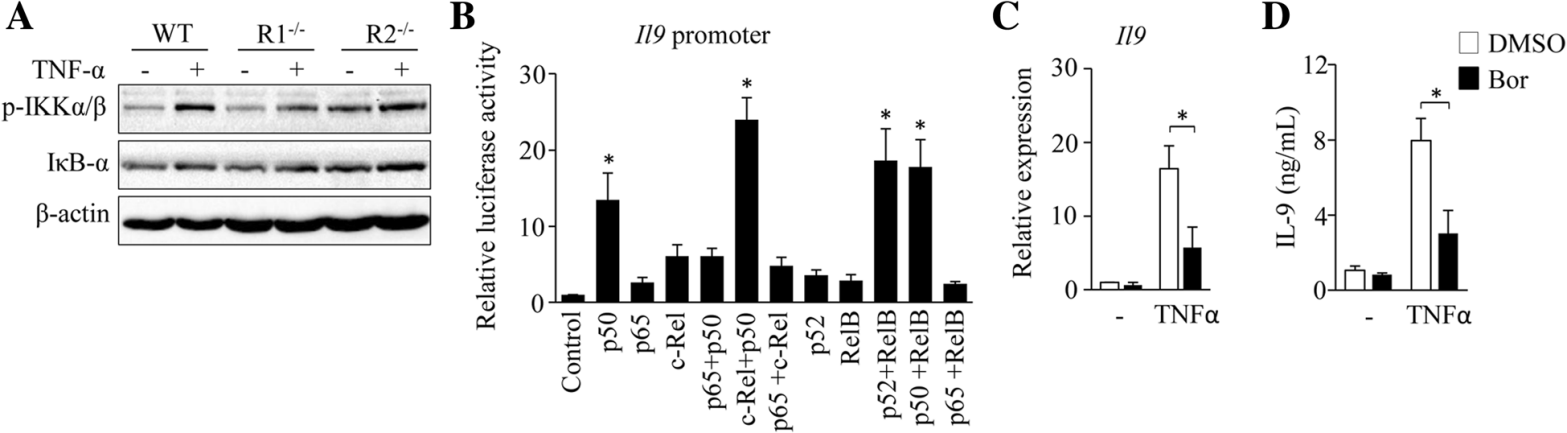
TNF-α enhances Th9 cell differentiation through NF-κB signaling pathway. (**a**) Naïve CD4^+^ T cells were cultured under Th9 polarizing conditions with or without addition of TNF-α for 3 h. Western-blots examined the protein levels of phosphorylated IKKα/β (p-IKKα/β) and IκB-α in cells. β-actin was used as a loading control. (**b**) 293 T cells were transiently transfected with vectors contained *Il9* promoter or empty vector, followed by transfecting with vectors expressing the indicated NF-κB molecules. Luciferase reporter assay showed NF-κB-dependent activation of *Il9* promoter in 293 T cells. (**c**, **d**) Naïve CD4^+^ T cells were cultured under Th9 polarizing conditions in the presence or absence of TNF-α with or without (DMSO) the addition of NF-κB inhibitor bortizomib (Bor) for 3 days. IL-9 expression was examined by qPCR (**c**) and ELISA (**d**). Data are representative of three (**a**) independent experiments or presented as mean ± SD of three (**b**-**d**) independent experiments. *P < 0.05

RNA-seq assay showed that TNF-α increased the expression of *Nfkb2* and *Traf6* in T cells (Fig. 5a), genes related to NF-κB signaling pathway. To further determine the role of TNF-α in the activation of NF-κB pathway in T cells, naïve CD4^+^ T cells were treated with TNF-α under Th9 polarizing conditions and cells were analyzed by Western-blots. TNF-α treatment increased the protein levels of p-IKKα/β in T cells (Fig. 6a), indicating that TNF-α activates NF-κB pathway during Th9 cell differentiation.

To determine the role of TNFR1 and TNFR2 in TNF-α-induced activation of NF-κB, naïve CD4^+^ T cells from WT, R1^−/−^ and R2^−/−^ mice were treated with TNF-α under Th9 polarizing conditions. R1^−/−^ CD4^+^ T cells treated with TNF-α have higher levels of p-IKKα/β than untreated controls (Fig. 6a); whereas TNF-α treatment exerted minor effects on the expression of p-IKKα/β in R2^−/−^ CD4^+^ T cells (Fig. 6a), indicating that TNF-α activates NF-κB pathway mainly through TNFR2.

We next explored the role of NF-κB pathway in TNF-α-induced Th9 cell differentiation. We first performed luciferase reporter assays to examine whether these NF-κB molecules could bind directly to *Il9* promoters and stimulate its expression. We found that p50, c-Rel-p50, p50-RelB and p52-RelB dimmers could bind to and activate *Il9* promoter (Fig. 6b). Bortezomib was used as an inhibitor of NF-κB pathway [11]. To further determine the role of NF-κB pathway in TNF-α-induced Th9 cell differentiation, Bortezomib was used during TNF-α-induced Th9 cell differentiation. Th9 cells treated by TNF-α plus bortezomib expressed lower levels of IL-9 mRNA and protein than those treated by TNF-α alone (Fig. 6c, d). These results indicated that TNF-α induces Th9 cell differentiation through NF-κB signaling pathway.

## Discussion

Tumor-specific Th9 cells are potential effector cells for adoptive therapy of human cancers [6]. Therefore, identifying factors that can stimulate Th9 cell development may have important clinical significance. Recently, TNF family cytokines OX40L, TL1A, and GITRL have been shown to promote Th9 formation and their antitumor effects [20–23]. However, the role of TNF-α in Th9 cell differentiation and their antitumor functions remains unknown. In this study, we found that TNF-α potently promotes Th9 cell differentiation and IL-9 production. In addition, TNF-α stimulates IL-9 expression in T cells at multiple time-points between Day 1 and Day 4 during Th9 cell differentiation. More importantly, the adoptive transfer of TNF-α-treated Th9 cells induces more potent antitumor effects than regular Th9 cells in mouse tumor models. Multiple mechanisms may be involved in the increased antitumor capability of TNF-α-treated tumor specific Th9 cells. The addition of TNF-α increases Th9 cell expression of IL-9, which is a mediator of antitumor immunity [6, 16]. TNF-α increases the expression of IL-2 in T cells [35] and the survival and proliferation of Th9 cells, which may prolong their persistence in vivo [6]. Furthermore, the high tumor-infiltrating capability of TNF-α-treated tumor-specific Th9 cells may also contribute to their antitumor efficacy. Thus, our data identify TNF-α as a new powerful inducer of Th9 cells.

TNF-α has two cell surface receptors TNFR1 and TNFR2 [24]. And T cells express both TNFR1 and TNFR2. In this study, we discovered that the blockade or depletion of TNFR2 in T cells abrogates the stimulatory effects of TNF-α on Th9 cell formation, indicating that TNFR2 downstream signaling is required for TNF-α-induced stimulation of Th9 cell formation. Compared to TNF-α/TNFR1 signaling, which has been shown to activate NF-κB, MAPK and caspase 8-mediated cell apoptotic pathways [36–38], the TNF-α/TNFR2 downstream signaling is not well characterized. Recent studies indicated that TNFR2 can interact with TRAF proteins TRAF1, 2 and 3 [39] and activate both the canonical and the noncanonical NF-κB signaling pathways [40, 41]. In this study, we found that TNF-α activates NF-κB signaling by increasing the expression of TRAF6 and p-IKKα/β in T cells. In addition, we discovered that TNF-α/TNFR2 signaling activates STAT5 pathway in T cells. And, blocking NF-κB or STAT5 by their specific inhibitors partially abrogates TNF-α-induced stimulation of Th9 cell formation. Therefore, TNF-α/TNFR2 signaling contributes to Th9 differentiation via two different mechanisms: the activation of (i) the transcription factor STAT5 and (ii) the TRAF6–NF-κB pathway. These two mechanisms may act synergistically to promote Th9 cell differentiation. Interestingly, published studies showed that STAT5 and NF-κB pathways are also involved in the stimulation of Th9 cell differentiation by other TNF family cytokines OX40L, TL1A, and GITRL [20–22], indicating that the TNF family cytokines may promote Th9 cell differentiation via some common signaling pathways.

TNF-α stimulates T cell to express IL-2, IL-5 and IL-9, which activate STAT5 pathway [42–44], suggesting the potential of TNF-α to indirectly activate STAT5 in Th9 cells. However, we found that TNF-α/TNFR2 activates STAT5 at the early stage of Th9 differentiation, and we did not observe increased expression of IL-2, IL-5 and IL-9 in T cells during that stage (Fig. 5A). These observations indicate that TNF-α/TNFR2 signaling can directly activate STAT5 during Th9 cell differentiation.

IL-5 and IL-13 are type-2 cytokines expressed primarily in Th2 and mast cells [45]. GATA3 is the master transcription factor for Th2 cells and controls the expression of Th2-derived cytokines, including IL-5 and IL-13 [45]. In this study, we found that TNF-α increases the expression of IL-5 and IL-13 in Th9 cells. However, TNF-α/TNFR2 signaling does not increase GATA3 expression, but activates the STAT5 and NF-κB pathways in T cells during Th9 cell differentiation. Interestingly, a previous study showed that STAT5 and NF-κB stimulate the expression of IL-5 and IL-13 in Th cells [46]. These observations suggest that TNF-α/TNFR2 signaling enhances the expression of IL-5 and IL-13 in Th9 cells through STAT5 and NF-κB pathways.

Cytokine milieu is the major determinant for Th cell differentiation [47]. In this study, we observed that TNF-α/TNFR2 signaling promotes Th9 cell differentiation and increases the antitumor capability of tumor-specific Th9 cells. However, previous studies also showed that TNFR2 signaling enhances the differentiation, expansion and function of Treg cells [48–50], suggesting inhibitory effects on antitumor immunity. These observations imply that the TNFR2 signaling promotes the differentiation and functions of not only Th9 cells but also Treg cells, which suggests that TNF-α/TNFR2 signaling may exert both beneficial and detrimental effects on tumor immunotherapy. Detailed mechanisms underlying the different role of TNF-α/TNFR2 signaling in the induction of Th9 cells and Treg cells need to be defined. Further studies will be necessary to investigate strategies of converting the TNF-α/TNFR2 signaling to the induction of Th9 cells but not Treg cells in tumor immunotherapy.

In summary, our study demonstrates that TNF-α potently promotes the induction of Th9 cells in vitro. And, the addition of TNF-α during Th9 cell differentiation increases T cell survival and proliferation. The adoptive transfer of TNF-α-treated Th9 cells induces potent therapeutic antitumor effects in mouse models. TNF-α drives Th9 cell differentiation via TNFR2 but not TNFR1, and TNF-α/TNFR2 signaling activates STAT5 and NF-κB pathways, which are required for TNF-α-induced Th9 cell differentiation. Our results identified TNF-α as a new powerful inducer of Th9 cells and clarified the molecular mechanisms underlying TNF-α-induced Th9 cell differentiation.
